# Off-Season Recovery Experiences Among Elite Women’s Rugby Union Players

**DOI:** 10.3390/bs16101840

**Published:** 2026-10-08

**Authors:** Stephen David Mellalieu, Paul Sellars, Owen Thomas, Rachel Arnold, Lee Moore

**Affiliations:** 1 School of Sport and Health Sciences, Cardiff Metropolitan University, Cardiff CF23 6XD, UK; psellars@cardiffmet.ac.uk (P.S.); othomas@cardiffmet.ac.uk (O.T.); 2 Department for Health, University of Bath, Bath BA2 7AY, UK; rsa24@bath.ac.uk (R.A.); ljm90@bath.ac.uk (L.M.)

**Keywords:** mental rest, psychological load, psychological detachment, workload management, recovery strategies, transition period, female athletes, elite sport

## Abstract

This study examined how elite women’s rugby union players perceived their off-season recovery experiences. An exploratory cross-sectional survey was completed by 140 elite women’s rugby union players (*M* age = 25.80, *SD* = 4.16) that captured global perceived readiness to train, recovery strategies, contextual demands, and preferred off-season structure. Quantitative data were analyzed descriptively, while responses to open-ended questions were explored using reflexive thematic analysis. Players reported a range of sport- and non-sport related demands during the off-season, including injury management, physical preparation, financial pressures, education and professional development, and external employment. Players reported a preferred off-season duration of 5.19 weeks on average (range 4–10 weeks), incorporating three broad components: a complete break from rugby, remote or personal training, and reintegration into the rugby environment. Overall, the findings indicate that elite women’s perceptions of off-season recovery reflect both rugby-related demands and wider occupational, educational and personal circumstances. The findings provide athlete-informed considerations for the design of off-season structures and identify areas for future prospective research.

## 1. Introduction

Elite rugby union is a physically and psychologically demanding sport requiring athletes to repeatedly perform high intensity running, collisions, and complex technical actions while coping with the physical and mental demands of prolonged competitive seasons (Cousins et al., 2023; Musgrave, 2021). These cumulative demands contribute to substantial physiological, biochemical, and psychological load, increasing players’ susceptibility to fatigue, injury, overtraining, and burnout (Cross et al., 2016; Dubois et al., 2017, 2020; Meeusen et al., 2013; Williams et al., 2017). Research has shown elevated injury risk is associated with high weekly training loads, large fluctuations in load, and extensive match exposure across a season (Cross et al., 2016; Williams et al., 2017). Concurrently, the experience of psychological fatigue and burnout has been documented in rugby players, with symptoms often linked to non-selection, excessive workload, or negative team environments (Cresswell & Eklund, 2006, 2007).

Although considerable attention has been paid to in-season workload management and acute recovery periods (cf. West et al., 2021), far less is known about how players recover from the cumulative demands of an entire competitive season. Most of the existing research has focused on short-term recovery following individual matches or weekly training microcycles (e.g., Shearer et al., 2015), with a particular emphasis on acute physiological responses and the role of sleep in recovery (Fullagar et al., 2015). Consequently, the off-season, which typically lasts approximately 4–6 weeks in elite rugby union, remains a comparatively underexplored phase of the annual training cycle, particularly with respect to the psychological and physical recovery processes that occur during this period (Mellalieu et al., 2023). Importantly, these processes may intersect in athletes’ experiences of recovery. Psychological recovery has been conceptualized as involving the alleviation of psychological load—the cumulative cognitive and emotional strain resulting from concurrent sport and life demands (Mellalieu et al., 2021). This load may be alleviated through experiences of mental rest, defined as a state of low cognitive demand free from performance expectations, which is achieved primarily through psychological detachment. Emerging literature on the psychology of recovery suggests that effective restoration requires both physical rest and psychological detachment, operationalized as a state of complete mental and emotional disengagement from sport-related thoughts, cues, and responsibilities (Balk et al., 2017; Eccles & Kazmier, 2019; Russell et al., 2019). Indeed, this form of cognitive distancing is a key protective factor against mental fatigue, stress accumulation, and impaired wellbeing (Sonnentag & Fritz, 2015).

Although psychological detachment has emerged as a central component of recovery, contemporary recovery theory suggests that successful restoration following prolonged performance demands is multifaceted (Sonnentag & Fritz, 2007; Sonnentag et al., 2022). Sonnentag et al. propose that recovery is facilitated through four complementary experiences comprising psychological detachment, relaxation, mastery experiences, and control over leisure time. Increasingly, these principles have been adopted within sport psychology, where effective recovery is recognized as extending beyond reductions in physical workload to include opportunities for athletes to psychologically disengage from performance demands, experience autonomy, reconnect with significant others, and engage in meaningful activities outside sport (Eccles & Kazmier, 2019). These complementary theoretical perspectives provide an important framework for understanding how elite rugby players recover during the off-season.

The off-season represents an important period in which athletes may have opportunities to distance themselves from the demands of the rugby environment. However, early evidence indicates that off-season experiences are neither uniform nor guaranteed (Mellalieu et al., 2023). Recent qualitative work with professional male rugby union players by Mellalieu et al. described three player-reported periods of decompression, cognitive detachment, and preparation for pre-season. Players’ accounts also highlighted rugby-related demands (e.g., contractual uncertainty and injury rehabilitation), life stressors (e.g., family responsibilities and financial pressures), health-related considerations (e.g., injury, pain and sleep), and differences in how players described managing the off-season. These findings provide useful contextual insight into how professional male rugby players may experience and organize the off-season, but they do not establish causal relationships between off-season experiences and subsequent recovery, wellbeing or readiness.

Although research on elite men’s rugby union has provided useful insight into off-season experiences, comparatively less empirical attention has been directed towards how elite women’s rugby union players experience and organize the off-season. Research in women’s elite sport has highlighted the importance of contextual factors such as employment, education, financial circumstances and competition structures (Culvin & Bowes, 2021; Heyward et al., 2025). These factors provide a relevant context for examining off-season recovery in women’s rugby, but the extent to which they shape players’ experiences within elite women’s rugby union has received comparatively limited empirical attention. There is limited research examining players’ perceptions of off-season demands, recovery strategies, readiness to return to training, and preferences regarding the structure and duration of the off-season. Understanding these experiences is therefore important for documenting how off-season recovery is perceived within the organizational, occupational and personal circumstances of elite women’s rugby union.

The present study therefore sought to provide a descriptive account of elite women’s rugby players’ perceptions of their most recent off-season, including their global perceived readiness to train, recovery strategies, contextual demands and views regarding the preferred structure and duration of an ideal off-season. In doing so, the study addresses a contextual knowledge gap concerning how off-season recovery is experienced within the organizational, occupational and personal circumstances of elite women’s rugby union. Specifically, the study aimed to:

1. Describe players’ perceived readiness to train following their most recent off-season.
2. Explore the sport-related, occupational and personal demands that players reported during the off-season.
3. Describe the recovery strategies reported by players and their perceived effectiveness.
4. Explore players’ recommendations regarding the preferred duration, structure and content of an ideal off-season.

## 2. Method

### 2.1. Participants

A purposive sample of *N* = 140 elite women’s rugby union players participated in this study (*M* age = 25.80 years, *SD* = 4.16). Participants were recruited from multiple national and competitive contexts to provide access to an elite women’s rugby population that is relatively difficult to access through probability-based sampling. Participants represented a range of elite playing contexts. Sixty-eight percent reported competing at international level, 15% in elite club competition, 5% academy participation, and 12% other elite playing contexts, including elite 7s or university level competition. Participants were therefore not exclusively international or women’s 15s international players, and the term “elite women’s rugby union players” is used throughout the manuscript to reflect the composition of the sample. The most represented nationalities were Canadian (19%), Welsh (14%), and French (13%). The primary domestic competitive landscapes where participants were based included England (23%), France (18%), and Wales (12%).

Participants reported an average playing experience of *M* = 4.70 years (*SD* = 3.23) at their current elite competitive level. The positional distribution encompassed both forwards (*n* = 71, 51%) and backs (*n* = 69, 49%). Specifically, the sample comprised back-three players (21%), half-backs (16%), back-row forwards (15%), locks (14%), centres (12%), props (12%), and hookers (10%). Participants also self-reported a range of professional-status categories: 32% reported they were fully professional (all income derived from rugby), 33% professional (rugby served as main income), 27% semi-professional, and 4% amateur (with 4% identifying as other). Fifty-seven percent of the sample reported maintaining employment or another occupational commitment outside rugby. These commitments included administration, the armed forces, education, sports medicine, fitness and coaching. Definitions of the professional-status categories can be found in the full survey instrument provided in Supplementary Material File S1.

### 2.2. Measures

#### 2.2.1. Demographic and Career Characteristics

Participants completed a self-report inventory capturing chronological age, nationality, country of competitive play, primary playing position, playing level, elite playing tenure, professional contractual status, and secondary employment commitments outside of rugby. These variables established the contextual framework for interpreting off-season recovery profiles.

#### 2.2.2. Perceived Readiness to Train

Global perceived readiness to train following the most recent off-season was assessed using a single-item, 8-point Likert-type scale. This item was intended to provide a practical, global indication of participants’ perceived readiness to resume training. Participants responded to the prompt: “*How ready to train (rested & recovered) were you following your latest off-season?*”. Responses were scored from 0 (*not ready to train*) to 7 (*fully ready to train*). Single-item indicators of athletic readiness demonstrate strong ecological validity and have been increasingly adopted within high-performance sport monitoring due to their practicality, low administrative burden, and ability to capture athletes’ perceptions of readiness and recovery status (Saw et al., 2016).

#### 2.2.3. Off-Season Demands

Contextual and systemic factors hindering recovery were captured by asking participants to list the off-season demands encountered and provide detailed open-ended responses outlining how these specific demands encroached upon their physical and psychological recovery.

#### 2.2.4. Recovery Strategies and Perceived Effectiveness

Players were asked to identify the behavioral, physical, and psychological strategies utilized during the off-season as recovery strategies through an open text response. Participants subsequently rated the perceived effectiveness of the strategies they reported on a 10-point scale ranging from 1 (*ineffective*) to 10 (*very effective*). Open-text responses were classified into broader recovery-strategy categories based on the content of participants’ responses. Where participants reported more than one strategy, each strategy was classified separately and linked to the corresponding perceived-effectiveness rating. Thus, the frequency of each category reflects the number of participants who reported using a strategy within that category, whereas effectiveness ratings reflect participants’ perceived effectiveness of the strategies they reported using.

#### 2.2.5. Perceived Off-Season Structure and Recommendations

Participants were asked to describe their perceptions of the impact of the current off-season structure in the country in which they played on their welfare and wellbeing. This was assessed through the open-ended question, “*Please provide your thoughts on the impact that the current off-season structure in the country where you play has upon your welfare and well-being*”. Players were also asked to indicate the duration (in weeks), that they considered appropriate for an off-season before returning to pre-season training. Finally, players responded to the open-ended question, “*If you could create the ‘ideal’ off-season to maximize physical and mental recovery what recommendations would you suggest?*”.

### 2.3. Procedures

The study formed part of a broader research project requested by World Rugby and International Rugby Players Association (IRPA) concerning off-season recovery among elite women’s rugby players (Mellalieu et al., 2025). World Rugby and IRPA facilitated access to the target player population and supported recruitment and data collection. Neither organization provided financial funding for the research. Prior to data collection, institutional ethical approval was granted by the institutional research ethics committee of the first author (Ethical approval no: STA-7699). Data collection spanned from 1 June 2024–31 March 2025 and included players participating within different competitive and geographical contexts. Participants were asked to reflect on their most recent designated off-season. Players were recruited through electronic distribution networks facilitated by player associations and governing bodies. Before accessing the survey, participants reviewed an information sheet which informed them of their ethical rights and provided explicit electronic informed consent. Participation was entirely voluntary, and anonymity was maintained throughout. No personally identifying information was collected, and all data were treated in strict compliance with institutional data management guidelines. World Rugby and IRPA subsequently reviewed the findings of the broader project report and provided comments and requests for additional detail. They did not review, write, edit or approve the academic manuscript and had no role in the analysis or interpretation of the academic findings. They also had no role in the decision to submit the academic manuscript or in the selection of the journal.

### 2.4. Survey Development and Administration

The survey was developed by the research team drawing on the objectives of the broader project and relevant literature concerning athlete recovery and off-season experiences. It was then reviewed by subject-matter experts, piloted and cognitively tested. The survey was administered in 4 languages (English, French, Spanish, and Japanese) and required approximately 10 min to complete. The pilot and cognitive-testing process was used to assess question clarity, response options and survey flow, following which minor wording and presentation revisions were made before administration. Translation procedures were determined during the broader project administration, and the present study did not independently evaluate measurement equivalence across language versions. Participant quotations presented in the manuscript were translated into English where necessary and were minimally edited for readability without altering their substantive meaning. The full survey instrument is provided in Supplementary Material File S1. Participants were not required to complete every open-ended question.

### 2.5. Data Analysis

The study used an exploratory cross-sectional survey design incorporating quantitative descriptive measures and qualitative open-ended responses. The quantitative and qualitative components were used to provide complementary descriptive information concerning players’ perceptions of their off-season experiences. Quantitative data were analyzed using SPSS Statistics (Version 28.0). Continuous variables, including age, global perceived readiness to train, perceived-effectiveness ratings and recommended off-season duration, were summarised using descriptive statistics. Depending on the distribution and level of measurement, these included means, standard deviations, medians, interquartile ranges and ranges. Categorical variables were summarised using frequencies and percentages. No inferential statistical tests were conducted because the purpose of the quantitative component was descriptive and exploratory. Analyses were conducted using available responses for each item, with missing responses excluded from the relevant item-level analysis and no imputation undertaken.

Responses to the open-ended survey questions were analyzed using reflexive thematic analysis following the six-phase approach described by Braun and Clarke (2019). This approach was selected because it provides a theoretically flexible interpretive method for identifying and developing patterns of meaning within qualitative data. The analysis was led by the second author (PS). Initial familiarisation involved reading and re-reading participants’ responses and making informal notes regarding issues relevant to the research questions. This was followed by line-by-line coding, with codes developed inductively from participants’ accounts of their off-season experiences. Coded extracts were subsequently organized into candidate themes by considering patterns across participants’ responses.

The initial themes and patterns were then independently reviewed and challenged by the first author (SM), who acted as a critical friend (Smith & McGannon, 2018). This process was intended to question assumptions, identify alternative interpretations and encourage consideration of contradictory or less readily accommodated accounts within the data. PS and SM subsequently worked collaboratively to review, refine and regroup codes and candidate themes. Themes were further reviewed for internal coherence, distinctiveness and consistency with the underlying participant accounts. Contradictory and negative cases were considered throughout this process rather than being excluded from the analysis.

The analysis was primarily inductive and was not undertaken to test or impose a pre-existing theoretical framework. The relevant literature was considered subsequently during interpretation and discussion of the findings rather than being used as a predetermined coding framework. The final themes and sub-themes therefore represent an interpretation of patterns of meaning within participants’ responses rather than a ranking of themes according to frequency or perceived importance. No qualitative analysis software was used.

### 2.6. Researcher Reflexivity

The qualitative analysis was conducted by two white, heterosexual academics (PS and SM), both of whom held doctoral degrees and had experience undertaking research within professional rugby or publishing research involving professional rugby populations. At the time of data collection, SM was also working as a mental health and player welfare professional within the male professional game in the UK. These experiences provided familiarity with the organizational and performance contexts in which participants’ experiences of the off-season were situated, while also presenting the potential for existing assumptions and professional experiences to influence interpretation.

Researcher positioning was therefore considered throughout the analysis. PS conducted the initial analysis, after which SM independently reviewed and challenged the initial patterns and themes. PS and SM subsequently worked collaboratively to review, refine and regroup the themes and sub-themes. This critical-friend process provided opportunities to question assumptions, consider alternative interpretations and attend to contradictory or less readily accommodated accounts within the data.

## 3. Results

### 3.1. Perceived Readiness to Train

Participants reported a mean global perceived readiness to train following their most recent off-season of *M* = 4.39 (*SD* = 1.46, 95% CI [4.14, 4.64]) on the 0–7 scale, where 0 represented “*not ready to train*” and 7 (*fully ready to train*). The median was 5.00 (*IQR* = 3.00–5.00), with scores ranging from 0 to 7. Of the 140 participants, *n* = 133 provided a valid response and *n* = 7 had missing data.

### 3.2. Player-Reported Off-Season Demands

Of the 140 participants, 130 responded to the off-season demands question. Reflexive thematic analysis of the open-ended responses identified seven themes describing sport-related, occupational and personal demands that featured in participants’ reported off-season experiences. These themes comprised injury management, physical preparation, rugby-specific administration, relocation, financial pressures, educational and professional development commitments, and external employment. The themes represent patterns of meaning identified across participants’ accounts and are not presented as a frequency ranking or as evidence that one demand was more important than another. Illustrative participant quotations are provided in Table 1. Responses reflected both sport and non-sport demands during the off-season. Respondents described a variety of factors that featured in their off-season experiences and opportunities for rest and detachment. Physiological demands included injury management and prescribed physical preparation. Players reported remaining engaged with sporting activities during the break, through rehabilitation appointments and related medical commitments. For example, one participant (P4) highlighted the relentless nature of these demands, stating, “It feels like the season never truly ends when you are jumping straight from the final match into mandatory rehab and daily conditioning blocks.” In addition, players described mandatory training activities during the off-season alongside rehabilitation commitments, with one player noting, “You are trying to fix your body while simultaneously hitting targets for the next pre-season, so the ‘break’ just becomes a different type of exhausting work.” (P117).

Players also reported organizational and career-related demands during the off-season. Rugby-specific administration and relocation were identified as themes, with players navigating contract negotiations, scheduling logistics, and the practical challenges of moving to join new clubs. These demands were described as involving ongoing logistical responsibilities during the off-season period. As one respondent (P45) noted, “Trying to organize a cross-country move and sort out club paperwork completely takes away any chance to mentally switch off.” Finally, the economic structure of the women’s game was described as contributing to dual-career demands, reported in the form of financial pressures, study/CPD commitments, and external employment requirements. This ‘dual burden’ was captured by a player who explained, “I cannot afford to just rest during the break, so my off-season is spent pulling extra shifts at work just to stay financially stable for the year ahead.” (P72).

### 3.3. Recovery Strategies and Perceived Effectiveness

Of the 140 participants, 133 provided responses to the open-ended recovery-strategy question. Participants reported a range of strategies that they perceived as useful during the off-season. These included holidays or travel, connecting with family and friends, active physical recovery, engagement in employment or study, hobbies and other non-rugby activities, and other personal recovery practices. Perceived-effectiveness ratings varied across the strategy categories (Table 2). Holidays or travel received the highest mean perceived-effectiveness rating *(M* = 8.33), followed by connecting with family and friends (*M* = 7.25) and active physical recovery (*M* = 7.25). Alternative employment or study, hobbies or other non-rugby activities, and other personal recovery practices received lower mean ratings.

### 3.4. Player-Reported Structural Factors and Systemic Barriers During the Off-Season

Of the 140 participants, 128 provided responses to the question concerning the perceived impact of current off-season structure on welfare and wellbeing. Players described a range of positive and negative perceptions of their current off-season structures. The principal facilitators and systemic barriers reported by the players are summarized in Table 3. Two recurring themes were identified as perceived facilitators: sufficient time to rest and recover, and a high degree of player autonomy over training. Players described uninterrupted time away from rugby as providing opportunities for rest, reduced perceived physical fatigue and psychological distance from rugby-related demands. Players also valued having the independence to select their own training types and locations during this period, describing this flexibility as supportive of their perceived recovery and sense of personal control. As one player explained, “Being able to decide where and how I train allowed me to reconnect with my normal life and actually look forward to rugby again” (P16).

Conversely, perceived negative impacts were associated with distinct systemic barriers. Inconsistent domestic and international season structures were reported to frequently create situations where different tournament schedules overlapped, leaving no clear gap for an off-season. Players described situations in which overlapping schedules were associated with limited time away from rugby, with these periods providing limited opportunity for perceived rest and recovery. Reflecting on this disruption, a respondent stated, “Because the club and international calendars don’t align, our break kept getting shifted until it completely vanished, leaving us with no time to heal” (P66).

Finally, for a portion of the cohort, players described circumstances in which the intended recovery period was constrained. This occurred when players reported having to utilize their sport-mandated rest period to fulfil external employment demands. Players attributed this reallocation of time away from rugby to financial circumstances as limiting their opportunities to access physical rest or psychological detachment and use the off-season for rest and recovery, with one player noting, “Instead of using the rest period to recover, I was on my feet working 40 h a week at my other job just to pay my bills” (P113).

### 3.5. Preferred Off-Season Duration and Player-Recommended Structure

Participants reported a preferred off-season duration of 5.19 weeks (*SD* = 2.09, 95% CI [4.84, 5.54]). The median was 5.00 (*IQR* = 4.00–6.00), with scores ranging from 4 to 10 weeks. All 140 participants provided a valid response. Responses to the ideal off-season question identified three broad components that players recommended as part of an optimal off-season structure (Table 4). In the first component: Complete break from training/rugby environment, players described the need for total detachment. This period was suggested to require a complete absence of training demands, communication from staff, and administrative responsibilities. Players described this component as an opportunity to recover from physical and psychological fatigue accumulated during the competitive season before any training resumed. In the second component: Remote or personal training, the focus shifted toward maintaining and rebuilding baseline physical conditioning. The distinguishing feature of this component was athlete autonomy. Rather than returning to supervised team environments, players recommended using individualized plans that could be completed independently and remotely. Players reported that individualized and remote training plans supported ongoing physical preparation while remaining away from the primary rugby environment. In the third component: Reintegration into club/rugby training environment, players recommended a gradual transition back into formal team operations. Rather than immediately returning to full-intensity pre-season training, this component was suggested to serve as a step-up period. It was suggested to allow for the progressive reintroduction of rugby-specific skills and collective training volumes, which players described as important for preparing for the demands of pre-season training.

## 4. Discussion

The present study explored elite women’s rugby union players’ perceptions of their most recent off-season, including global perceived readiness to train, reported recovery strategies, off-season demands and preferred off-season structure. The findings indicate that players’ experiences of the off-season reflected a combination of rugby-related demands and wider occupational, educational, financial and personal circumstances. Players also described several strategies that they perceived as useful, including time away from rugby, social connection and active physical recovery. In addition, responses to the ideal off-season question identified three broad player-recommended components: a complete break from rugby, remote or personal training, and reintegration into the rugby environment. These findings should be interpreted as descriptive accounts of players’ perceptions rather than evidence of causal relationships between off-season structures, recovery processes and subsequent outcomes. The findings are nevertheless consistent with broader recovery literature emphasising opportunities for psychological detachment, relaxation, social connection and control over activities undertaken away from sport (Sonnentag & Fritz, 2007; Eccles & Kazmier, 2019).

### 4.1. Player-Recommended Components of an Ideal Off-Season

One notable finding was the consistency with which participants described three broad components of an ideal off-season: an initial period away from rugby, a period of individually managed or remotely supported preparation, and subsequent reintegration into the rugby environment. The first component involved a clear period of separation from rugby-related training, communication and administrative demands. The second involved maintaining or rebuilding physical preparation through individually managed or remotely supported training, while remaining outside the immediate team environment. The final component involved a gradual return to collective rugby activities and preparation for the demands of the forthcoming season.

Our findings for elite female rugby union players also closely align with those from Mellalieu et al.’s (2023) qualitative investigation of professional male rugby union players. The convergence between the present findings and those reported in male rugby suggests that some features of the preferred off-season experience may be shared across elite rugby populations. Specifically, previous research has highlighted the value of opportunities to step away from performance demands and engage in activities that provide distance from sport-related cues (Balk et al., 2017; Eccles & Kazmier, 2019). In the present study, players similarly emphasised the value of time away from rugby before returning to individually managed physical preparation and, subsequently, the rugby environment.

The present findings therefore contribute to a view of the off-season as more than a singular period of reduced training load. Instead, players’ recommendations incorporated both opportunities for separation from rugby and a gradual return towards preparation for future performance. This interpretation is broadly consistent with contemporary recovery models that emphasise restoration and preparation as important aspects of athlete functioning (Kellmann & Beckmann, 2017; Kellmann et al., 2018). However, the three components identified in the present study should be regarded as an athlete-informed description of preferred off-season organization rather than a universal or empirically established sequence through which recovery necessarily progresses.

### 4.2. Psychological Detachment and Time Away from Rugby

Participants frequently described time away from rugby, including holidays, travel and social activities, as useful aspects of their off-season experience. These findings are consistent with theoretical accounts that identify psychological detachment from work or performance-related demands as one potentially important recovery experience (Sonnentag, 2012; Sonnentag & Fritz, 2015). Although the present data cannot establish psychological detachment as a mechanism or mediator of recovery, participants’ descriptions suggest that opportunities to disengage from rugby-related responsibilities and activities were valued alongside physical rest and other forms of recovery. This interpretation is also consistent with sport-specific literature suggesting that recovery may involve experiences beyond simply reducing physical training load (Eccles & Kazmier, 2019). Across participants’ responses, the value of these activities appeared to relate to their capacity to provide temporary distance from rugby-related thoughts, responsibilities and identities. Furthermore, activities associated with time away from rugby may encompass several different recovery experiences. Specifically, holidays and travel may provide opportunities for relaxation, whereas time spent with family and friends may provide opportunities for social affiliation (Sonnentag & Fritz, 2007). These distinctions are relevant because players did not describe recovery simply in terms of physical inactivity; rather, their accounts included a range of activities that allowed them to step away from the immediate demands and routines of rugby.

The prominence of psychological detachment within the player accounts is consistent with broader recovery research emphasising the importance of temporary disengagement from work-related demands (Sonnentag, 2012; Sonnentag & Fritz, 2015). However, in the present study, psychological detachment is best understood as an interpretive concept that helps contextualise players’ descriptions rather than as a demonstrated recovery mechanism. The study did not directly measure psychological detachment or examine whether it mediated relationships between time away from rugby and recovery outcomes. Instead, the findings indicate that players valued opportunities to create psychological and physical distance from rugby during the off-season, alongside opportunities for relaxation, social connection and other non-rugby activities.

### 4.3. Dual-Career Demands and the Absence of an Off-Season

Although opportunities for psychological detachment and physical recovery were evident in players’ accounts, the present study also identified a range of contextual demands that featured in players’ accounts of the off-season. These included external employment, study and continuing professional development, relocation, financial pressures, and ongoing rugby-related administration. For some players, these demands occupied periods that they otherwise regarded as an opportunity to rest and recover. This was particularly apparent in accounts from players who combined rugby with employment or other occupational commitments, illustrating that the designation of a period as an “off-season” did not necessarily mean an absence of competing demands. The role of employment and study in players’ recovery experiences was, however, not uniformly negative. Players described non-rugby activities in different ways, with some accounts suggesting that work, study or professional development provided structure, purpose, or an opportunity to focus attention on areas of life beyond rugby. In other accounts, employment or educational commitments were experienced as competing with opportunities for rest, particularly where such commitments were financially necessary or difficult to reconcile with the timing of the off-season. This distinction is important because the findings do not support treating all non-rugby activity as a barrier to recovery. Rather, players’ accounts suggest that the circumstances, demands and degree of choice associated with these activities were relevant to how they experienced the off-season.

This interpretation is consistent with research in occupational psychology distinguishing between activities that may involve continued effort or obligation and activities that provide opportunities for relaxation, detachment and recovery (Sonnentag et al., 2017). From this perspective, employment, study and other responsibilities may represent continuing demands during a period intended for recovery, although the present data cannot establish their effects on recovery outcomes. Indeed, some forms of non-rugby activity may simultaneously provide personally meaningful experiences, a sense of accomplishment, or opportunities to develop resources outside sport. Consequently, the findings are better understood as illustrating the different ways in which players’ non-rugby commitments intersected with their recovery experiences rather than demonstrating that such activities necessarily impair recovery.

The findings are also consistent with broader literature identifying career uncertainty, financial pressures and competing life demands as relevant psychosocial considerations for elite athletes (Kuettel & Larsen, 2020). Research examining professional women’s football has similarly highlighted the importance of understanding elite sport within the wider occupational and social circumstances in which athletes work and live (Culvin & Bowes, 2021). The present findings extend these observations to women’s rugby union by illustrating how employment, education and financial circumstances were incorporated into players’ descriptions of the off-season. For some participants, the period intended to provide recovery from rugby was also a period in which they needed to maintain employment or address other occupational responsibilities.

### 4.4. Autonomy as a Facilitator of the Off-Season Experience

Another prominent feature of players’ accounts concerned the value placed on autonomy and flexibility during the off-season. Participants described preferences for having some control over where, when and how they undertook training, conditioning and recovery activities. This was particularly evident in relation to the remote or personal training component of the ideal off-season, where players valued maintaining physical preparation away from the immediate rugby environment.

The importance placed on autonomy is broadly consistent with self-determination theory, which identifies autonomy as a basic psychological need associated with motivation and psychological functioning (Ryan & Deci, 2020). The findings can also be considered alongside the recovery experience of control described by Sonnentag and Fritz (2007), whereby individuals have some degree of control over what activities they undertake and when and how they undertake them. Similarly, Eccles and Kazmier (2019) emphasised the importance of opportunities for athletes to step away from environments in which their activities are controlled by sport and engage in activities beyond their sporting role. In the present study, players’ preferences for flexibility and self-direction can be understood as consistent with these perspectives. However, autonomy should be interpreted as a valued feature of players’ reported off-season experiences rather than as a demonstrated causal mechanism of recovery. The present study did not directly assess autonomy, psychological need satisfaction or wellbeing using validated measures, and its cross-sectional design does not permit conclusions regarding causal relationships. Instead, the findings suggest that players valued having sufficient freedom to determine how they balanced rest, non-rugby activities and physical preparation according to their individual circumstances.

This preference for autonomy is also relevant to the three broad components identified by participants in their ideal off-season: a complete break from the training/rugby environment, remote or personal training, and reintegration into the rugby environment. These should be understood as player-recommended components rather than universally applicable sequential stages. From an applied perspective, the findings suggest that off-season planning may benefit from providing protected periods of rest alongside appropriate flexibility in training and recovery activities, allowing players some choice over how they manage their transition away from and back into organized rugby. Such approaches are athlete-informed considerations rather than established interventions.

### 4.5. Practical Implications

The findings provide several athlete-informed considerations for organizations, coaches and practitioners responsible for planning off-season structures. These should be regarded as considerations arising from players’ reported experiences rather than interventions whose effectiveness has been established by the present study. First, organizations may wish to consider protecting a clearly defined period during which players have minimal or no rugby-related training, administrative and communication requirements. Participants’ accounts suggest that the opportunity to create meaningful distance from rugby was valued during this period. Second, communication boundaries may be considered when establishing protected rest periods. Clear expectations regarding essential contact, administrative requirements and the timing of pre-season communication may help players understand when they are expected to remain engaged with rugby and when they can reasonably disengage. Third, where physical preparation is required during the off-season, players’ preference for individualized or remotely supported training could be considered. Providing flexibility regarding the location, timing and format of conditioning may allow players to maintain preparation while retaining greater control over their off-season activities. Fourth, off-season planning should take account of employment, education and financial circumstances. For players who require external employment, simply designating a period as an “off-season” may not create meaningful time for recovery if that period is occupied by work or other unavoidable commitments. Finally, players should be involved in the design and review of off-season structures. Consultation could identify the practical barriers experienced by different groups of players and help organizations distinguish between activities that players experience as meaningful non-rugby engagement and those that represent unavoidable occupational or financial demands.

These considerations require prospective evaluation. Future research should examine whether off-season structures are associated with changes in recovery, readiness, wellbeing, injury or performance before specific practices are considered evidence-based interventions.

### 4.6. Limitations and Future Directions

Several limitations should be considered when interpreting the findings. First, the cross-sectional design and retrospective self-report approach mean that the study captures participants’ perceptions of their most recent off-season rather than prospectively observed changes in recovery. Participants may therefore have been affected by recall bias, and the findings cannot establish causal relationships between off-season structures, recovery strategies, wellbeing or subsequent performance. Because readiness, recovery strategies, perceived effectiveness and off-season experiences were reported within the same self-report survey, common-method and self-report biases also cannot be excluded. Second, global perceived readiness to train was assessed using a single self-report item. Although this provided a practical indication of participants’ overall perception of being rested and ready to train, it did not independently assess psychological and physical recovery and should not be interpreted as a validated multidimensional recovery measure. Future research could combine global readiness ratings with validated psychological measures, physiological indicators and behavioral measures such as sleep. Third, no validated measure of wellbeing or objective recovery was administered. The wellbeing findings therefore represent participants’ perceptions rather than measured changes in wellbeing. Future prospective research should incorporate multidimensional measures of psychological wellbeing, recovery experiences and physical recovery alongside repeated readiness assessments.

Fourth, participants were purposively recruited to access an elite women’s rugby population that is relatively difficult to study using probability-based approaches. While this approach was appropriate for the exploratory purpose of the study, it limits the generalisability of the findings beyond the participating population. Indeed, a formal response rate could not be calculated because recruitment occurred through multiple electronic distribution networks and a complete denominator of players approached was not available. The multinational sample also incorporated variation in competitive calendars, professional status and employment circumstances, which may shape individual off-season experiences. The survey did not record the exact interval between the end of each participant’s off-season and survey completion; therefore, recall intervals could not be standardized. Fifth, professional-status categories were self-reported and may not be directly comparable across countries or rugby systems. The present study did not conduct formal subgroup analyses by professional status, employment, playing level, country or hemisphere. Such comparisons would be valuable in future research but would require sufficiently large samples and clearly operationalised categories. Sixth, the three components identified in responses to the ideal off-season question were player recommendations rather than longitudinally observed stages. The study therefore cannot determine whether players actually progress through these components in a consistent order or whether particular off-season structures are associated with better recovery outcomes.

Finally, the bespoke survey instrument was designed for the broader research project and, although it enabled the collection of both quantitative and qualitative information from a relatively large elite sample, it does not provide the psychometric breadth of a comprehensive recovery assessment. Recovery is multidimensional and includes physical restoration as well as psychological experiences such as relaxation, psychological detachment from sport-related demands, social connection and control over activities undertaken during leisure time (Sonnentag & Fritz, 2007; Eccles & Kazmier, 2019; Sonnentag et al., 2022). Future studies should build on these findings using validated measures and prospective designs to examine how off-season timing, duration, structure, employment circumstances, psychological detachment and player autonomy relate to dimensions of recovery and wellbeing over time.

## 5. Conclusions

This exploratory study provides a descriptive account of elite women’s rugby union players’ perceptions of their off-season experiences. Players described a range of rugby-related, occupational, educational, financial and personal demands that featured in their accounts of opportunities for rest and engagement during the off-season. Players also identified several strategies that they perceived as useful, including time away from rugby, social connection and active physical recovery. Responses concerning an ideal off-season identified three broad player-recommended components: a complete break from rugby, remote or personal training, and reintegration into the rugby environment. The findings highlight the value of considering players’ wider occupational and personal circumstances when designing off-season structures. They provide athlete-informed considerations for future practice and policy while highlighting the need for prospective research using validated psychological, physical and behavioral measures to determine how different off-season structures relate to recovery, wellbeing and readiness to train.

## Figures and Tables

**Table 1:** Themes, Definitions, Manifestations and Example Quotes of Player-Reported Off-Season Demands Impacting Recovery.

Higher-Order Theme	Description	Core Manifestations	Example Participant Quote
Injury Management	Ongoing medical and clinical obligations related to physical injury.	Navigating mandatory rehabilitation, and consultations without true detachment.	*“I’m stuck doing rehab and physio for my knee, still going to the venue to get treatment, I don’t feel as though I have had a break, it feels like Groundhog Day.”*
Physical Preparation	Prescribed physiological training loads required during the break.	Managing mandatory pre-season or maintenance training volumes, limiting opportunities for complete physical rest.	*“Even though it is supposed to be our off-season, we are limited from getting real physical rest because of the conditioning and gym work, and mandatory rugby sessions we still have to complete.”*
Rugby-Specific Administration	Logistical and contractual tasks associated with career management.	Dealing with contract negotiations, scheduling logistics, and administrative communications with new or existing clubs.	*“The admin load is exhausting, between navigating the constant comms required for sorting out my contract and managing the scheduling logistics for international 7s and 15s.”*
Relocation	Geographical transition demands linked to club playing contracts.	Navigating the domestic or international logistical demands of moving cities or countries to join new club environments.	*“The biggest demand for me has been moving overseas to play, which has forced me to completely reorganize my entire life to make the move happen.”*
Financial Pressures	Economic stressors stemming from the sport’s professionalization status.	Managing financial anxieties directly linked to lower athletic financial remuneration in the women’s game.	*It’s a lot of stress trying to manage day-to-day life when facing these constant financial headaches, which are a result of the lower pay we get.”*
Educational and Professional Development Commitments	Dual-career developmental tasks outside of athletic performance.	Balancing concurrent academic degrees or continuous professional development (CPD) requirements.	*“Trying to advance my career outside of sport means I am constantly forced into balancing my Uni study commitments alongside rugby.”*
External Employment	Vocational commitments required to generate a livable secondary income.	Undertaking supplementary work commitments outside rugby during the off-season.	*“There is no time to relax during the break because you have to match your outside commitments alongside training demands just to secure a large enough wage to live off.”*

*Note.* Themes represent analytically developed patterns of meaning within participants’ responses and are not ranked according to frequency or assumed to reflect relative thematic importance.

**Table 2:** Recovery Strategies Used During the Off-Season and Perceived Effectiveness.

Recovery Strategy	*n* (%)	Mean	95% CI	SD	Median	IQR	Range
Holidays and travel	21 (15%)	8.33	7.63–9.03	1.53	8	7.50–9.00	7–10
Connecting with family and friends	29 (20%)	7.25	6.07–8.43	3.10	8	6.00–9.25	3–10
Active physical recovery (e.g., light training)	51 (36%)	7.25	6.98–7.52	0.96	7.5	6.75–8.00	6–8
Alternative employment or study	21 (15%)	6.43	4.90–7.96	3.36	8	3.50–9.00	2–10
Engaging in hobbies or non-rugby activities	29 (20%)	5.56	4.28–6.84	3.36	5	3.00–8.00	1–10
Other personal recovery practices	65 (46%)	5.33	4.54–6.12	3.21	4	3.50–6.50	3–9

*Note. n* and % indicate the number and percentage of participants who reported using a strategy within each category. Effectiveness ratings were provided only for strategies reported as used and are presented on a 1–10 scale (1 = ineffective; 10 = very effective). Percentages may not sum to 100% because participants could report multiple recovery strategies. All participants who reported using a strategy provided an effectiveness rating; therefore, the *n* values for strategy use also represent the valid effectiveness-rating sample for each category. 95% CI = 95% confidence interval for the mean perceived-effectiveness rating.

**Table 3:** Player-Reported Structural Factors and Systemic Barriers During the Off-Season.

Category	Theme	Structural Manifestation
Principal Facilitators	Sufficient rest time	Allocation of consecutive weeks with no training or team commitments to allow physical and mental recovery
	Athlete autonomy	Flexibility to choose personal conditioning activities and recovery locations away from club oversight
Systemic Barriers	Inconsistent season structures	Overlapping domestic, international, and regional schedules that disrupt the scheduling of a clear break
	Insufficient time off	Shortened recovery windows due to extended competitive seasons or early pre-season start dates
	Loss of off-season recovery opportunity	Elimination of rest periods due to unavoidable external work or dual-career commitments

**Table 4:** Player-Recommended Components and Key Requirements for an Ideal Off-Season.

Component	Focus	Key Structural Requirements
Complete Break	A period of separation from rugby-related training and demands	Absence of mandatory training schedules, communication from coaching staff, and rugby administrative tasks
Remote or Personal Training	Autonomous physical preparation	Individualized, self-guided conditioning plans completed away from the primary club environment
Reintegration	Transition back to collective training structures	Step-up in physical load, introduction of rugby-specific skills, and re-entry into the team environment

## Data Availability

The raw data supporting the conclusions of this article will be made available by the authors on request.

## Supplementary Materials

The following supporting information can be downloaded at https://www.mdpi.com/article/10.3390/bs16101840/s1. File S1: Survey Questionnaire.
