# Supplementary material for: Off-Season Recovery Experiences Among Elite Women’s Rugby Union Players
Source: Behav Sci (Basel). 2026 Oct 8;16(10):1840. doi: 10.3390/bs16101840 (PMC13701317; doi:10.3390/bs16101840)
Supplement: Supplementary file 1 [file behavsci-16-01840-s001.zip › behavsci-4463845-supplementary.pdf]

File S1. Survey Questionnaire

1. Year of birth:
2. Nationality:
3. Current Country where you play rugby:
4. Primary playing position:
5. Please select the statement which best describes your current employment status:
    - Professional rugby player (income exclusively from playing)
    - Professional rugby player (main income from playing)
    - Semi-professional rugby player (some income from playing/some income from other employment)
    - Amateur rugby player (main income from other employment)
    - Other ____

6. If you earn income outside of playing professional rugby throughout the season or in the off-season please tell us:
    - The job title(s):
    - Average hours per week worked in the job(s) during the season:
    - Average hours per week worked in the job(s) during the off-season:
    - How much annual paid leave you dedicated to rugby overall throughout the season? (e.g. 6 days)
    - How much annual unpaid leave you dedicated to rugby overall throughout the season?
    - How much annual paid leave you dedicated to rest and recovery in the off-season?
    - How much annual unpaid leave you dedicated to rest and recovery in the off-season?
    - Please feel free to add any additional comments:

7. If you are undertaking any study commitments throughout the season or in the off-season please tell us:
    - The name of the study programme (e.g. MA Sports Management):
    - Average hours per week spent on your study commitments during the season:
    - Average hours per week spent on your study commitments during the off-season:
    - Please feel free to add any additional comments:
    ____

8. Please select the statement which best describes your current rugby status
    - Academy player
    - Squad member for first XV
    - Regular starter for first XV
    - International (Test level) player
    - Other ____

9. How many years have you been playing rugby at your current level?

10. Thinking about the start of your most recent pre-season, please rate the extent to which you felt you were ready to train following the off-season period.
(0 - not ready to train to 7 - fully ready to train)

11. How would you describe your physical and mental well-being in the most recent off-season?
(Please consider how you felt in this time period, if there were any changes in your mental and physical well-being, and the impact of your mental and physical well-being upon your life)

12. Please list the main demands that impacted upon your mental and physical recovery during the most recent off-season? (e.g. non-rugby commitments - work/study/career)

13. Please tell us about the strategies you used to recover in the last off-season mentally and physically to be ready for the most recent pre-season - Write a strategy you used in the box provided and then rate the effectiveness of that strategy to help you recover (0 = not effective, 10 = very effective)

14. Please provide your thoughts on the impact that the current off-season structure in the country where you play has upon your welfare and well-being.
(Please consider the importance of the off-season, current length etc.)

15. How long do you think the off-season period should be for you to recover fully ready for your next season?

16. Based on your experiences as a player, If you could create the 'ideal' off-season to maximise physical and mental recovery what recommendations would you suggest?
(Please include aspects such as length, content, amount of contact with your club, training requirements)
